# The Physiological Significance of TRP and Piezo Channels as Physical Stimulus Sensors in Brown Adipocytes

**DOI:** 10.3390/cells15030293

**Published:** 2026-02-04

**Authors:** Kunitoshi Uchida, Mari Iwase

**Affiliations:** 1 Laboratory of Functional Physiology, Department of Environmental and Life Sciences, School of Food and Nutritional Sciences, University of Shizuoka, Shizuoka 422-8526, Japan; imari@u-shizuoka-ken.ac.jp; 2 Graduate School of Integrated Pharmaceutical and Nutritional Sciences, University of Shizuoka, Shizuoka 422-8526, Japan

**Keywords:** TRP channel, Piezo channel, Ca^2+^ signal, energy metabolism, differentiation, thermogenesis, brown adipocytes

## Abstract

**Highlights:**

**What are the main findings?**

- This review highlights Ca^2+^-permeable ion channels, particularly TRPV2 and Piezo1, as important regulators of brown adipocyte differentiation and thermogenesis.
- Recent evidence supports the concept of a stage-dependent shift in ion channel function during brown adipocyte differentiation, which may involve changes in channel sensitivity and regulatory mechanisms.

**What are the implications of the main findings?**

- These findings clarify the role of Ca^2+^ signaling in brown adipocyte responses to physiological stimuli.
- Cation-channel-mediated Ca^2+^ signaling plays an important role in regulating thermogenesis and metabolic homeostasis.

**Abstract:**

Most transient receptor potential (TRP) channels are Ca^2+^-permeable non-selective cation channels that function as polymodal receptors activated by a wide variety of stimuli, including natural compounds such as pungent substances, physical stimuli, lipids, intracellular signaling molecules, and ions. Their physiological roles are diverse, including sensory perception, ion transport, and intracellular signaling. Similarly, Piezo channels, which are also Ca^2+^-permeable non-selective cation channels, are activated by mechanical stimuli such as membrane stretching and contribute to touch sensation, blood flow regulation, and bladder-filling sensation, among other functions. While research on non-selective cation channels in relation to energy metabolism has primarily focused on TRP channels expressed in primary afferent neurons, studies over the past decade have revealed the important roles of TRP and Piezo channels in brown adipocytes. In this review, we highlight evidence regarding the contributions of TRPV2 and Piezo1 to brown adipocyte differentiation and thermogenesis and briefly summarize recent advances regarding other TRP channels expressed in brown adipocytes. Furthermore, we propose a conceptual framework in which a “modal shift” in TRP/Piezo channels, defined as developmental stage-dependent changes in their functional properties, may contribute to the regulation of brown adipocytes’ functions.

## 1. Introduction

### 1.1. Brown and White Adipose Tissues

Adipose tissue is a highly plastic organ that dynamically adapts to environmental, nutritional, and neural cues to maintain systemic energy homeostasis [1]. In mammals, adipose tissue is broadly categorized into white adipose tissue (WAT) and brown adipose tissue (BAT), with an additional population of recruitable brown-like adipocytes, called beige (or brite) adipocytes, emerging within subcutaneous WAT depots under specific physiological conditions [2]. While WAT’s primary function is to store energy, BAT is specialized for energy expenditure. A defining functional property of BAT is Uncoupling protein 1 (UCP1)-dependent non-shivering thermogenesis, which dissipates the proton gradient generated by oxidative phosphorylation as heat [3,4]. This thermogenesis is initiated by sympathetic nervous system activation, typically in response to cold exposure or gastrointestinal chemical stimuli such as pungent compounds, leading to noradrenaline release and subsequent β-adrenergic receptor signaling in brown adipocytes [5]. The high mitochondrial density and dense sympathetic innervation of BAT make this tissue particularly well suited for stimulus-responsive energy dissipation. Beige adipocytes share key functional features with classical brown adipocytes, including inducible UCP1 expression and thermogenic capacity, although their developmental origin and transcriptional profiles are different from brown adipocytes [6,7]. Recruitment of beige adipocytes is triggered by sustained cold exposure or pharmacological β3-adrenergic receptor activation, highlighting the importance of environmental and neural inputs in shaping and determining adipose tissue phenotypes.

The presence of metabolically active BAT in adult humans, once considered minimal, has been firmly established by fluorodeoxyglucose positron emission tomography–computed tomography (FDG-PET/CT) imaging studies [8,9,10]. Histological and transcriptomic analyses have identified UCP1-positive adipocytes and heterogeneous adipocyte populations with brown- and beige-like characteristics in human adipose depots [2,11]. Importantly, BAT abundance and activity are inversely correlated with adiposity-related parameters, including body fat mass and body mass index (BMI) [8,12], highlighting the contribution of thermogenic adipocytes to whole-body energy balance. Consistent with these observations, experimental models demonstrate that sustained activation of BAT thermogenesis through prolonged cold exposure or β3-adrenergic stimulation increases BAT mass and reduces adiposity [13], whereas genetic impairment of thermogenic capacity, such as in UCP1-deficient mice, leads to obesity in animals under chronic high-fat feeding conditions [14]. Collectively, these findings indicate that BAT functions as a highly stimulus-responsive metabolic tissue, and that disruption of its adaptive thermogenic capacity can contribute to metabolic imbalance. The general features of white, brown, and beige adipocytes have been described elsewhere; here, attention is directed to stimulus-responsive mechanisms in brown and beige adipocytes, particularly those involving Ca^2+^ signaling and ion channels.

### 1.2. The Mechanism of Thermogenesis in Brown and Beige Adipocytes

Intracellular Ca^2+^ signaling has been implicated in the regulation of adipocyte differentiation and thermogenic activation. In brown adipocytes, β3-adrenergic receptor signaling promotes mitochondrial uncoupling via UCP1, resulting in dissipation of the proton gradient across the inner mitochondrial membrane and the conversion of chemical energy into heat [15]. Long-chain fatty acids generated by lipolysis and redox-dependent modifications of UCP1 have been proposed as important contributors to this process [16,17]. Along with these processes, Ca^2+^ signaling has been shown to influence adipocyte differentiation. In preadipocytes, increases in intracellular Ca^2+^ concentration ([Ca^2+^]_i_) suppress differentiation through calcineurin-dependent pathways and inhibition of insulin signaling, as demonstrated in 3T3-L1 cells [18,19,20]. These findings indicate that Ca^2+^ signaling may exert distinct effects depending on the differentiation states of adipocytes.

In differentiated brown and beige adipocytes, Ca^2+^ dynamics appear to participate in the regulation of thermogenesis. Changes in [Ca^2+^]_i_ have been linked to UCP1 expression and whole-body energy expenditure [21]. In addition, ATP-dependent Ca^2+^ cycling mediated by Sarco/endoplasmic reticulum Ca^2+^-ATPase 2b (SERCA2b) and Ryanodine receptor 2 generates UCP1-independent heat in beige adipocytes [22], whereas membrane hyperpolarization that limits Ca^2+^ influx impairs adrenergic thermogenesis in brown adipocytes [23]. More recently, adrenergic stimulation has been shown to promote the formation of a mitochondrial complex containing the Mitochondrial Ca^2+^ uniporter (MCU), its regulatory component (Essential MCU regulatory element; EMRE), and UCP1, thereby enhancing mitochondrial Ca^2+^ uptake and supporting uncoupled respiration [24]. Despite these advances, how Ca^2+^ influx is initiated, coordinated, and tuned in adipocytes under physiological conditions remains poorly understood. This suggests that Ca^2+^-permeable ion channels, including transient receptor potential (TRP) and Piezo channels, may act as upstream regulators connecting physiological stimuli that induce Ca^2+^ signaling to adipocyte differentiation and thermogenesis.

### 1.3. Transient Receptor Potential Channels

TRP channels comprise a large family of Ca^2+^-permeable ion channels, most of which are non-selective [25]. Structurally, TRP channels share a conserved architecture consisting of six transmembrane domains (TM1-TM6) and a pore-forming loop between TM5 and TM6, with both N- and C-termini located in the cytosol [26]. In mammals, the TRP superfamily comprises six subfamilies: TRPV (Vanilloid), TRPC (Canonical), TRPM (Melastatin), TRPML (Mucolipin), TRPP (Polysystin), and TRPA (Ankyrin), encompassing a total of 28 channels [27,28]. Functionally, TRP channels act as polymodal sensors that are activated by diverse physical and chemical stimuli, including temperature, mechanical force, endogenous lipids, oxidants, ions, and bioactive compounds [29,30]. Among these, several TRP channels exhibit temperature-dependent activation within physiologically relevant ranges; they are collectively referred to as thermo-sensitive TRP channels (thermo-TRPs). Eleven thermo-TRPs have been identified in mammals, primarily belonging to the TRPV, TRPM, TRPA, and TRPC subfamilies. TRPV1, TRPV2, and TRPM3 respond to elevated temperatures, whereas TRPM8, TRPA1, and TRPC5 are activated by cool or cold stimuli, although the cold sensitivity of mammalian TRPA1 remains controversial. TRPV3, TRPV4, TRPM2, TRPM4, and TRPM5 are activated by warm temperatures [31].

Importantly, TRP channels are expressed not only in sensory neurons and skin but also in a wide range of non-sensory tissues that are not directly exposed to rapid environmental temperature changes. In such contexts, TRP channels are thought to function as integrators of cellular signaling downstream of physiological stimuli. Notably, multiple TRP channels are expressed in tissues involved in energy intake and expenditure, including the hypothalamus, gastrointestinal tract, peripheral sensory neurons, liver, and adipose tissue [32,33,34]. Growing evidence indicates that TRP channels in adipocytes contribute to the regulation of differentiation, thermogenesis, and metabolic function, largely through Ca^2+^-dependent mechanisms [35,36].

### 1.4. Piezo Channels

Piezo channels were identified in 2010 as mechanically activated non-selective cation channels through an RNAinterference screen targeting stretch-evoked currents in mammalian cells [37]. Structural analyses have revealed that Piezo proteins assemble as large trimeric complexes forming a three-bladed propeller-like architecture with a peripheral curved membrane footprint that is well suited for sensing mechanical forces [38]. Two members of the Piezo family are present in mammals: Piezo1 and Piezo2. Although both channels act as mechanotransducers, their expression patterns and physiological roles differ. Piezo1 is mainly expressed in non-sensory tissues and functions as a shear-stress sensor in vascular endothelium and a detector of pressure changes in organs such as the bladder [39,40]. In contrast, Piezo2 is predominantly expressed in sensory neurons, including dorsal root ganglion neurons, and Merkel cells, and plays essential roles in touch sensation, proprioception, and mechanical pain [41,42].

Piezo channels are increasingly recognized as regulators of diverse biological processes, such as vascular development, bone formation, immune function, and respiratory control [43]. Piezo1, in particular, integrates extracellular mechanical cues with Ca^2+^ signaling to influence cell fate, proliferation, and differentiation. Emerging evidence indicates that Piezo1 is expressed in metabolic tissues, including adipose tissue, and may participate in adipocyte differentiation, adipose remodeling, and systemic metabolic regulation, suggesting additional roles for Piezo channels beyond somatosensation.

While our previous reviews summarized the roles of TRP channels in systemic energy metabolism and thermoregulation, less attention has been paid to how the functional stimuli and signaling roles of these channels change across adipocyte developmental stages [35,44]. In this review, we focus on the concept of A developmental-stage-dependent “modal shift” in TRPV2 and Piezo1 functions in brown adipocytes, highlighting how identical Ca^2+^-permeable channels can exert distinct effects on differentiation versus thermogenic activation depending on expression levels, activation stimuli, and cellular expression levels.

## 2. Functional TRP/Piezo Channels in Brown Adipocytes

Although TRP channels have been well studied in primary afferent neurons, particularly TRPV1, in the vagal sensory pathways involved in dietary sensing, increasing evidence indicates that several TRP and Piezo channels are also expressed in brown adipose tissue and brown adipocytes, and their physiological functions have been elucidated (Table 1). In the following sections, we focus on the physiological roles of TRPV2 and Piezo1 in brown adipocytes, which have been examined in detail by our group and others, and we also summarize recent findings on other TRP channels expressed in brown and beige adipocytes.

### 2.1. TRPV2

TRPV2 is a Ca^2+^-permeable non-selective cation channel that is activated by noxious heat (>52 °C) and mechanical stimuli such as membrane stretch. TRPV2 is expressed in neurons, the heart, and immune cells and shows particularly high expression in brown adipose tissue [51]. In undifferentiated brown adipocytes, functional TRPV2 expression is low but increases during differentiation, suggesting that TRPV2 plays important roles in differentiated brown adipocytes.

In BAT from systemic TRPV2 knockout (TRPV2KO) mice, brown adipocytes and lipid droplets are enlarged, and whitening-like morphological changes are observed. Consistent with these phenotypes, thermogenic genes including UCP1 are downregulated, whereas genes associated with lipid storage tend to be upregulated, suggesting an imbalance in BAT energy metabolism. Physiologically, TRPV2KO mice show a greater reduction in core body temperature during 4 °C cold exposure, without changes in locomotor activity or sympathetic nerve activity, compared with wild-type mice. Moreover, the β3-adrenergic agonist–induced increase in interscapular BAT temperature is blunted in TRPV2KO mice under anesthesia [51].

In contrast, whole-body oxygen consumption, locomotor activity, and food intake do not differ between TRPV2KO and wild-type mice under standard housing conditions. This observation suggests that alterations in TRPV2-dependent thermogenic phenotypes may not be reflected in basal energy expenditure under standard housing conditions [51].

**Figure 1:**
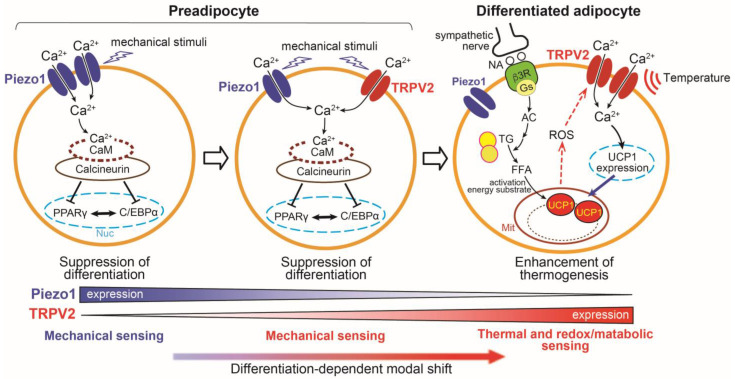
Physiological roles of TRPV2 and Piezo1 channels in brown adipocytes. In brown preadipocyte, the mechano-sensitive channel Piezo1 is highly expressed, and its activation suppresses the differentiation via the calcineurin pathway. During differentiation, Piezo1 expression decreases, whereas the expression of another mechano-sensitive channel, TRPV2, is upregulated and has been reported to negatively regulate differentiation. In differentiated brown adipocytes, upregulated TRPV2 is required for full β3-adrenergic induction of thermogenic gene expression and contributes to non-shivering thermogenesis. In addition, TRPV2 activity may be modulated by tissue temperature and redox signaling, including methionine oxidation associated with mitochondria-derived reactive oxygen species (ROS) during thermogenic activation. Together, these observations form the basis of a proposed functional “modal shift” in TRPV2 and Piezo1 across the adipocyte life cycle. CaM; Calmodulin. PPARγ; peroxisome proliferator-activated receptor γ, C/EBPα; CCAAT/enhancer-binding protein α, NA; Noradrenaline, AC; Adenylyl cyclase, TG; triglyceride, FFA; Free fatty acid, Nuc; Nucleus, Mit; Mitochondria.

In isolated mouse primary brown adipocytes, the induction of UCP1 gene expression by treatment with β-adrenergic agonists is also almost abolished in brown adipocytes from TRPV2KO mice. Similarly, induction of UCP1 expression and non-esterified fatty acid release by an adenylyl cyclase (AC) activator, forskolin, is impaired in TRPV2-deficient cells, and chelation of intracellular Ca^2+^ by BAPTA-AM suppresses the β-adrenergic receptor activation-induced upregulation of UCP1. These observations support the idea that TRPV2-mediated Ca^2+^ influx is required for full β-adrenergic induction of thermogenic gene expression, although the precise position of TRPV2 relative to AC/cAMP signaling remains to be determined and could be influenced by additional factors such as changes in redox state or local temperature, as discussed below. In addition, other TRPV2-activating inputs, including mechanical stimulation (membrane stretch), bioactive lipids such as lysophospholipids and endocannabinoids [64,65,66,67], or growth factor–dependent membrane recruitment of TRPV2, cannot be excluded as contributing mechanisms [68,69]. These findings suggest that TRPV2 in differentiated brown adipocytes contributes to non-shivering thermogenesis (Figure 1) [51].

When obesity is induced by feeding a high-fat diet (HFD), TRPV2KO mice develop more severe obesity than wild-type mice. While systemic deletion of TRPV2 does not exclude the contribution of other tissues, this phenotype suggests that TRPV2-dependent thermogenic mechanisms contribute to whole-body energy expenditure. Human brown adipocytes are thought to resemble beige adipocytes, which are induced within white adipose depots upon cold exposure, rather than classical rodent brown adipocytes [70]. Cold-induced recruitment of beige adipocytes is reduced in TRPV2KO mice [51], implying that TRPV2 may also play important roles in human brown/beige adipocytes.

As noted above, TRPV2 is already detectable in preadipocytes, although its expression is low and markedly increases during adipocyte differentiation. When a TRPV2 agonist is applied from the onset of differentiation, adipocyte differentiation is suppressed. TRPV2 activation reduces the expression of Peroxisome proliferator-activated receptor γ (PPARγ), a key transcription factor for adipocyte differentiation, without suppressing brown adipocyte-specific genes such as PPARγ coactivator 1 α (PGC-1α) and PR domain containing 16 (PRDM16). Mechanical stimulation likewise inhibits brown adipocyte differentiation, and pharmacological inhibition of TRPV2 suggests that this effect is mediated, at least in part, by TRPV2 activation. Ca^2+^ influx is known to inhibit white adipocyte differentiation through Ca^2+^/calmodulin-dependent activation of calcineurin, and differentiation of brown adipocytes is similarly suppressed by TRPV2 activation. This inhibition is partially reversed by calcineurin inhibitors [52]. Consistent with this idea, recent work showed that selective TRPV2 inhibitors derived from natural coumarin enantiomers reverse TRPV2 agonist-induced suppression of brown adipocyte differentiation, providing pharmacological support for TRPV2-mediated Ca^2+^ entry as a negative regulator of adipogenesis [71]. Thus, TRPV2 exerts stage-dependent functions during the brown adipocyte differentiation process (Figure 1).

Although endogenous activators of TRPV2 in brown adipocytes remain incompletely defined, several candidate stimuli have been proposed, including membrane stretch, bioactive lipid mediators, and Insulin growth factor 1 (IGF-1)–induced membrane recruitment of TRPV2. Recent work has further shown that oxidation of methionine residues at positions 528 and 607 markedly lowers the temperature threshold for TRPV2 activation toward physiological ranges [72]. Consistent with these findings, treatment with a methionine oxidant (chloramine-T) activates TRPV2 at ~30 °C in TRPV2-expressing HEK293T cells and in differentiated brown adipocytes, and potentiates the expression of thermogenesis-related genes [53]. These observations raise the possibility that post-translational modifications, such as methionine oxidation, contribute to TRPV2 sensitivity to intracellular and/or tissue temperature and redox conditions. Given that mitochondrial reactive oxygen species increase during elevated thermogenic activity, such redox changes could be linked to metabolic demand and participate in the modulation of TRPV2-dependent thermogenic responses (Figure 1). However, it should be noted that chloramine-T has low specificity, and its effects on thermogenic gene expression may reflect oxidative stress signaling beyond TRPV2-specific activation. Further clarification is required to determine whether changes in thermogenic gene expression are attributable to TRPV2-specific signaling or secondary responses associated with oxidative stress.

### 2.2. Piezo1 Channel

Mechanical stimulation during the differentiation of brown adipocytes suppresses differentiation, and functional TRPV2 expression is low in brown preadipocytes. These observations suggested that other mechano-sensitive ion channels may be involved. In mouse brown preadipocytes, Piezo1 is strongly expressed, whereas its functional expression decreases during the progressive differentiation, in contrast to TRPV2. Activation of Piezo1 suppresses brown adipocyte differentiation, similar to the effect of TRPV2 activation. Piezo1 activation also reduces the expression of PPARγ, without suppressing brown adipocyte–specific genes such as PGC-1α and PRDM16. This suppression was completely reversed by a calcineurin inhibitor (FK506), and the enhancement of calcineurin activity by the Piezo1 agonist Yoda-1 is completely abolished by silencing Piezo1 in preadipocytes. Together, these findings suggest that Piezo1-mediated increase in intracellular Ca^2+^ activates calcineurin, thereby inhibiting adipocyte differentiation (Figure 1) [61]. Conversely, the knockdown of Piezo1 via siRNA promoted differentiation under static culture conditions without the application of external mechanical stimulation or a Piezo1 agonist. Although these in vitro conditions do not quantitatively mimic the mechanical environment of adipose tissue in vivo, this observation supports the notion that Piezo1 acts as a sensor for basal or low-level mechanical inputs, including cell movement, during adipocyte differentiation.

Piezo1 is also expressed in brown adipose tissue, and Piezo1 protein is detectable in brown adipocytes containing lipid droplets [61], indicating the possibility that Piezo1 also exerts functional roles in differentiated brown adipocytes. Its expression is elevated in the adipose tissue of obese mice. Adipocyte-specific Piezo1KO mice exhibit insulin resistance and impaired adipose remodeling when fed with a HFD, together with adipocyte hypertrophy and increased inflammatory tone in perigonadal white adipose tissue (pgWAT) [62]. In addition, analyses of adipocyte-specific Piezo1 knockout mice show that activation of Piezo1 in differentiated white adipocytes induces secretion of fibroblast growth factors (FGFs), which act on progenitor cells to promote their differentiation [63]. Lack of Piezo1 in differentiated white adipocytes impaired preadipocyte-to-adipocyte differentiation under HFD feeding, a defect accompanied by adipocyte hypertrophy, elevated white adipose tissue inflammation, and reduced insulin sensitivity [63]. Although direct evidence in brown adipocytes is completely lacking, it is possible that a similar Piezo1-dependent FGF signaling mechanism, from differentiated brown adipocytes to preadipocytes, could also operate in BAT.

### 2.3. Other TRP Channels

Recent studies implicated TRPV1 in adipocyte biology. TRPV1 expression, activated by dietary capsaicin and heat, has been reported in the 3T3-L1 adipocyte cell line, and its activation induces a Ca^2+^ influx that suppresses adipogenesis, likely via calcineurin-dependent mechanisms [45]. TRPV1 activation also enhances thermogenic gene expression and promotes browning during adipocyte differentiation [48], although TRPV1 inhibition has been reported to increase PGC-1α expression, indicating context-dependent regulation. Beyond effects on differentiation, TRPV1 appears linked to thermogenic capacity. Capsaicin treatment elevates intracellular and mitochondrial Ca^2+^ handling and stimulates both UCP1-dependent and ATP-dependent thermogenesis through β-adrenergic and Ca^2+^-dependent pathways [49]. TRPV1 has also been detected in a subset of brown preadipocytes in mice. A population of vascular smooth muscle–derived adipocyte progenitors expressing TRPV1 expands in response to cold and differentiates into thermogenic adipocytes [46]. Moreover, combined loss of TRPV1 and UCP1 results in severe impairment of BAT mitochondrial respiration and thermogenesis, suggesting that TRPV1 may contribute to mitochondrial Ca^2+^ regulation in brown adipocytes. Collectively, these findings support the role of TRPV1 in the regulation of adipocyte differentiation and thermogenic function, although the physiological modes of TRPV1 activation in adipocytes require further clarification.

TRPV4 is expressed in both brown and white adipose tissues [54]. In the white adipocyte cell line 3T3-F442A, knockdown of TRPV4 induces the expression of thermogenic genes such as UCP1, whereas TRPV4 activation leads to phosphorylation of ERK and JNK and suppresses thermogenic gene expression. Consistent with these findings, TRPV4 knockout mice are resistant to HFD-induced obesity, and pharmacological inhibition of TRPV4 promotes browning of white adipose tissue [54]. In contrast, adipose-specific overexpression of TRPV4 has also been reported to protect mice against diet-induced obesity and to promote white fat browning via activation of the AKT pathway. This phenotype was accompanied by reduced adipose inflammation and improved insulin sensitivity [55]. Together, these observations suggest that the metabolic impact of TRPV4 signaling depends on cellular context and signaling state. Notably, TRPV2KO and TRPV4KO mice display opposite phenotypes in response to high-fat feeding, indicating that the role of Ca^2+^ signaling in adipocytes is highly channel- and context-dependent, including the timing of channel activation during differentiation or metabolic challenge.

TRPM8 has been reported to be expressed in mouse brown adipose tissue. TRPM8 activation by menthol increases UCP1 expression in brown adipose tissue via protein kinase A activation [58]. In addition, the activation of TRPM8 in human white adipocyte cell lines induces the browning of white adipocytes [60]. More recently, menthol pretreatment of the skin is shown to enhance cold tolerance and mitigate cold injury in mice via TRPM8-dependent activation of BAT thermogenesis, further supporting the physiological role of TRPM8 in adaptive heat production [59]. These findings raise the possibility that, at least in vivo, TRPM8 expressed in sensory neurons may contribute to the modulation of BAT thermogenesis.

Collectively, these findings suggest that multiple TRP channels contribute to the regulation of the adipocyte phenotype and thermogenesis. These seemingly opposing phenotypes likely reflect differences in cell type, differentiation stage, stimulus intensity and duration, and systemic and adipocyte-autonomous effects.

## 3. Conclusions and Perspectives

Most TRP and Piezo channels are Ca^2+^-permeable, non-selective cation channels that regulate cellular excitability and elicit downstream signaling responses through Ca^2+^ influx. In brown adipocytes, the expression and functional contributions of these channels change during development. Piezo1 is highly expressed in preadipocytes, where it is thought to sense mechanical inputs arising from cell movement, volume changes, or lipid accumulation, thereby acting as a negative regulator of differentiation through Ca^2+^/calcineurin signaling. As differentiation proceeds, Piezo1 expression declines, whereas TRPV2 expression increases, and TRPV2 similarly suppresses differentiation when activated during the early stages, at least in part via the calcineurin-dependent pathway. In mature brown adipocytes, upregulated TRPV2 functionally contributes to β3-adrenergic induction of thermogenic gene expression and non-shivering thermogenesis. Although TRPV2 likely detects mechanical input during early differentiation in a manner analogous to Piezo1, accumulating evidence suggests that TRPV2 in differentiated adipocytes may become responsive to changes in the redox state and tissue temperature, conditions that intensify when metabolic demand and heat production increase. Together, these observations support the idea that brown adipocyte function is regulated by sequential shifts in the dominant activation cues and signaling roles of mechanically sensitive channels, such as Piezo1 and TRPV2, rather than by static channel functions across all developmental stages.

Emerging evidence further suggests that TRPV2 activity in differentiated brown adipocytes may be modulated by post-translational mechanisms, such as methionine oxidation, potentially enabling TRPV2 to integrate metabolic or oxidative signals with thermogenic responses during sympathetic activation. However, it remains unclear whether such modifications occur in vivo during non-shivering thermogenesis and whether they are required for TRPV2-dependent Ca^2+^ signaling. In contrast, Piezo1 appears to act primarily during the early lineage stages and could exert additional indirect effects on adipose tissue remodeling. The distinct stimuli detected by Piezo1 and TRPV2, together with their different periods of activity during adipocyte development, form the basis for a developmental-stage-dependent functional “modal shift” model for TRP and Piezo channel signaling in brown adipocytes (Figure 1).

In addition to TRPV2 and Piezo1 channels, several TRP channels, including TRPV1, TRPV4, and TRPM8, have been reported to be involved in adipocyte differentiation, browning, and thermogenic regulation. TRPV1 has been linked to adipocyte browning and intracellular Ca^2+^-related thermogenic signaling, whereas TRPV4 and TRPM8 differentially modulate metabolic and thermogenic responses. Other TRP channels, such as TRPV3, TRPM4, TRPA1 and TRPC5, are likewise expressed in white and/or brown adipocytes and have been implicated in the regulation of processes including adipocyte differentiation. Along with stage-dependent changes in Piezo1 and TRPV2 activity, additional TRP channels likely act cooperatively to regulate brown adipocyte function. Together, these channels likely form a broader regulatory network that supports thermogenic and metabolic responses in brown adipose tissue.

Despite these advances, several key questions remain unresolved. First, the mechanisms that regulate the developmental-stage-dependent expression and functional engagement of TRPV2 and Piezo1 remain largely unknown. The transcriptional, post-transcriptional, and/or epigenetic programs that establish these shifts in channel availability and responsiveness are yet to be defined, and elucidating these processes would substantially strengthen the mechanistic foundation of the modal shift model. Second, the mechanism by which TRPV2 positively regulates the thermogenic functions of mature brown adipocytes remains unclear. While TRPV2-mediated Ca^2+^ influx has been linked to the induction of thermogenic gene expression, it is unclear whether TRPV2 primarily acts as a permissive Ca^2+^ entry pathway or whether its activity is tuned by physiological inputs associated with thermogenesis, such as local temperature elevation or changes in the cellular redox state. In particular, whether temperature-dependent gating and methionine oxidation of TRPV2 occur in vivo during non-shivering thermogenesis and whether these processes are necessary for TRPV2-dependent Ca^2+^ signaling remain poorly understood. Moreover, whether TRPV2 activity in vivo contributes not only to thermogenic activation but also to the regulation of brown adipocyte differentiation in a stage-dependent manner remains to be determined. Finally, it is also unclear whether Piezo1-mediated Ca^2+^ signaling in vivo exerts sustained effects on adipocyte differentiation or adipose tissue remodeling, including early lineage stages.

Future studies clarifying how TRP and Piezo channels are activated under physiological and pathophysiological conditions, and how their functions shift during adipocyte development, will further advance our understanding of thermogenic regulation. Given the widespread expression and diverse physiological roles of TRP and Piezo channels, their potential as therapeutic targets for metabolic diseases requires careful consideration of tissue specificity and safety. In particular, tissue-specific (e.g., Ucp1-Cre or Adipoq-Cre mice) and developmental-stage-dependent (e.g., Myf5-Cre mice) deletions of TRP and Piezo channels in adipocytes are essential to distinguish cell-autonomous effects from systemic or developmental contributions and to test the proposed modal shift model. The relevance of the proposed modal shift framework to human brown or beige adipocytes remains to be established, as functional evidence in human systems is currently limited. Moreover, even in mouse models, the physiological significance and regulatory mechanisms of this framework are not yet fully defined. Addressing these issues is essential for improving our understanding of thermogenic regulation and its potential relevance to metabolic diseases.

## Figures and Tables

**Table 1:** Expression and roles of TRP/Piezo channels in brown and beige adipocytes.

Channel	Thermo- and Mechano-Sensitivity	Other Activating Stimuli/Modulators	Expression in Adipocytes and Adipose Tissues	Stage-Dependent Roles in Brown and Beige Adipocytes
TRPV1	Heat (>42 °C)	capsaicin, proton, shanshool, allicin, camphor, resiniferatoxin, vanillotoxin, 2-APB, propofol, anandamide, arachidonic acid metabolic products (by lipoxygenases), NO, extracellular cation, phosphorylation by PKC and PKA	Mouse WAT [45], human WAT [45], vascular smooth muscle cells [46], Mouse BAT [47], 3T3-L1 cell [45,48,49], HB2 cell [50]	3T3L1 cell: browning ↑ Brown adipocyte: thermogenesis ↑
TRPV2	Heat (>52 °C) Mechanical stimulation	probenecid, 2-APB, cannabidiol, lysophosphatidylcholine, lysophosphatidylinositol, methionine oxidation	Mouse BAT [51,52], Mouse brown adipocytes [53]	Mature brown adipocyte, BAT: thermogenesis ↑ Differentiating brown adipocyte: differentiation ↓
TRPV4	Warm (>27–41 °C) Hypoosmolality Mechanical stimulation	4α-PDD, bisandrographolide, citric acid, arachidonic acid metabolic products (by epoxygenases), anandamide, GSK1016790A, phosphorylation by PKC	Mouse WAT [54,55,56], Human WAT [57], 3T3-F422A cell [54]	3T3-F422A cell, WAT: browning ↑
TRPM8	Cool (<27 °C)	menthol, icilin, eucalyptol, PIP_2_	Mouse BAT [58,59], Mouse WAT [60], Human white adipocyte [60]	Brown adipocyte: thermogenesis ↑ White adipocyte: browning ↑
Piezo1	Mechanical stimulation	yoda-1	Mouse Brown adipocytes [61], Mouse WAT, BAT [62,63]	Brown preadipocyte: differentiation ↓ WAT: browning ↑

“↑” indicates facilitation or an increase, and “↓” indicates impairment or a decrease.

## Data Availability

No new data were created or analyzed in this study.

## Abbreviations

The following abbreviations are used in this manuscript:

TRP	Transient receptor potential
TRPV	Transient receptor potential vanilloid
BAT	Brown adipose tissue
WAT	White adipose tissue
UCP1	Uncoupling protein 1
FDG-PET/CT	Fluorodeoxyglucose positron emission tomography–computed tomography
BMI	Body mass index
[Ca^2+^]_i_	Intracellular Ca^2+^ concentration
SERCA2b	Sarco/endoplasmic reticulum Ca^2+^-ATPase 2b
MCU	Mitochondrial Ca^2+^ uniporter
EMRE	Essential MCU regulatory element
TM	Transmembrane domains
KO	Knockout
AC	Adenylyl cyclase
HFD	High-fat diet
PPARγ	Peroxisome proliferator-activated receptor γ
PGC-1α	PPARγ coactivator 1 α
PRDM16	PR domain containing 16
IGF-1	Insulin growth factor 1
FGF	Fibroblast growth factors
CaM	Calmodulin
C/EBPα	CCAAT/enhancer-binding protein α
NA	Noradrenaline
TG	Triglyceride
FFA	Free fatty acid
Nuc	Nucleus
Mit	Mitochondria
